# The relationship between gastric emptying, plasma cholecystokinin, and peptide YY in critically ill patients

**DOI:** 10.1186/cc6205

**Published:** 2007-12-21

**Authors:** Nam Q Nguyen, Robert J Fraser, Laura K Bryant, Marianne J Chapman, Judith Wishart, Richard H Holloway, Ross Butler, Michael Horowitz

**Affiliations:** 1 Department of Gastroenterology and Hepatology, Royal Adelaide Hospital, North Terrace, Adelaide, South Australia, 5000; 2 Discipline of Medicine, University of Adelaide, Royal Adelaide Hospital, Adelaide, South Australia, 5000; 3 Investigation and Procedures Unit, Repatriation General Hospital, Daw Road, Adelaide, South Australia, 5000; 4 Department of Anaesthesia and Intensive Care, Royal Adelaide Hospital, Adelaide, South Australia, 5000; 5 Centre for Paediatric and Adolescent Gastroenterology, Children, Youth and Women's Health Service, Adelaide, South Australia, 5000

## Abstract

**Background:**

Cholecystokinin (CCK) and peptide YY (PYY) are released in response to intestinal nutrients and play an important physiological role in regulation of gastric emptying (GE). Plasma CCK and PYY concentrations are elevated in critically ill patients, particularly in those with a history of feed intolerance. This study aimed to evaluate the relationship between CCK and PYY concentrations and GE in critical illness.

**Methods:**

GE of 100 mL of Ensure^® ^meal (106 kcal, 21% fat) was measured using a ^13^C-octanoate breath test in 39 mechanically ventilated, critically ill patients (24 males; 55.8 ± 2.7 years old). Breath samples for ^13^CO_2 _levels were collected over the course of 4 hours, and the GE coefficient (GEC) (normal = 3.2 to 3.8) was calculated. Measurements of plasma CCK, PYY, and glucose concentrations were obtained immediately before and at 60 and 120 minutes after administration of Ensure.

**Results:**

GE was delayed in 64% (25/39) of the patients. Baseline plasma CCK (8.5 ± 1.0 versus 6.1 ± 0.4 pmol/L; *P *= 0.045) and PYY (22.8 ± 2.2 versus 15.6 ± 1.3 pmol/L; *P *= 0.03) concentrations were higher in patients with delayed GE and were inversely correlated with GEC (CCK: *r *= -0.33, *P *= 0.04, and PYY: *r *= -0.36, *P *= 0.02). After gastric Ensure, while both plasma CCK (*P *= 0.03) and PYY (*P *= 0.02) concentrations were higher in patients with delayed GE, there was a direct relationship between the rise in plasma CCK (*r *= 0.40, *P *= 0.01) and PYY (*r *= 0.42, *P *< 0.01) from baseline at 60 minutes after the meal and the GEC.

**Conclusion:**

In critical illness, there is a complex interaction between plasma CCK, PYY, and GE. Whilst plasma CCK and PYY correlated moderately with impaired GE, the pathogenetic role of these gut hormones in delayed GE requires further evaluation with specific antagonists.

## Introduction

In health, cholecystokinin (CCK) and peptide YY (PYY) are important humoral mediators of nutrient-induced small intestinal feedback, which regulates gastric emptying (GE) and energy intake [1-5]. In response to the presence of nutrients (particularly fat and protein) in the small intestine, CCK and PYY are released in a load-dependent manner from enteroendocrine cells, predominantly in the proximal small intestine for CCK and the distal small intestine for PYY [5-8]. CCK has also been reported to mediate the initial postprandial release of PYY [9,10]. In healthy humans, exogenous administration of CCK and PYY is associated with relaxation of the proximal stomach, inhibition of antral motor activity, stimulation of contractions localised to the pylorus, slowing of GE [1,2,4,7,11,12], and a reduction in energy intake [3,4,13-16]. CCK antagonists have been shown to increase GE and energy intake in humans [17-19]. The effects of PYY antagonism on GE in humans, however, are unknown. Furthermore, both plasma CCK and PYY concentrations are elevated in patients with chronic nutrient deprivation, malnutrition, and anorexia nervosa [20-22], conditions that are known to be associated with a high prevalence of delayed GE [23,24].

Impaired gastric motor function and associated feed intolerance occur in up to 50% of critically ill patients and can adversely affect both morbidity and mortality [25,26]. Whilst the mechanisms underlying delayed GE in critical illness remain poorly defined, exaggerated inhibitory feedback on GE arising from the interaction of nutrients with the small intestine is likely to be important [27]. For example, in response to duodenal nutrient, there is a greater degree of antral hypo-motility, pyloric hyperactivity [27], and exaggerated release of both CCK and PYY in critically ill patients [28,29]. Furthermore, the CCK and PYY responses are substantially greater in those patients who have feed intolerance [28,29]. In the fasted state, there is an increase in plasma concentrations of hormones that slow GE, such as CCK and PYY, and a decrease in hormones that may accelerate GE, such as ghrelin [28-30]. The effects of exogenous CCK and PYY on gastric motility are also comparable to the motor disturbances in both the proximal and distal stomach observed in critically ill patients [27,31,32].

Whereas the above evidence supports a potential role for both CCK and PYY in the mediation of enhanced nutrient-induced enterogastric feedback during critical illness, the relationships between plasma CCK and PYY concentrations and GE in critical illness have hitherto not been evaluated. This study was designed to examine the following hypotheses: (a) slow GE is associated with elevated plasma concentrations of CCK and PYY, and (b) GE is a determinant of postprandial concentrations of CCK and PYY in the critically ill.

## Materials and methods

### Subjects

Studies were performed prospectively in 39 unselected critically ill patients (24 males; 55.8 ± 2.7 years old) who were admitted to a level-3 intensive care unit (ICU) between May 2005 and November 2006. Any patient at least 17 years old was eligible for inclusion if he or she was sedated, mechanically ventilated, and able to receive enteral nutrition. Exclusion criteria included any contraindication to passage of an enteral tube; a history of gastric, oesophageal, or intestinal surgery; recent major abdominal surgery; evidence of liver dysfunction; administration of prokinetic therapy within 24 hours prior to the study; and a history of diabetes mellitus. All patients were receiving an insulin infusion according to a standard protocol, which was designed to maintain the blood glucose concentration between 5.0 and 7.9 mmol/L [27-29,31]. Written informed consent was obtained from the next of kin for all patients prior to enrolment into the study. The study was approved by the Human Research Ethics Committee of the Royal Adelaide Hospital and performed according to the National Health and Medical Research Council guidelines for the conduct of research on unconscious patients.

### Study protocol and techniques

Critically ill patients were studied in the morning, after a minimum 8-hour fast. All patients were sedated, with either propofol or a combination of morphine and midazolam, throughout a minimum of 24 hours prior to the study. The type of sedation was determined by the intensivist in charge of the patient and did not influence patient selection. In all patients, a 14- to 16-French gauge Levin nasogastric feeding tube (Pharma-Plast, Lynge, Denmark) was already *in situ *in the stomach, as part of clinical care, and the correct position of the feeding tube was confirmed radiologically prior to commencing the study.

GE was measured by a ^13^C-octanoate breath test, with the patient in the supine position and the head of the bed elevated to 30°. Gastric contents were initially aspirated and discarded, and then 100 mL of liquid nutrient meal (Ensure™; Abbott Australia, Kurnell, Australia) containing 106 kcal with 21% of fat and labelled with 100 μL of^13^C-octanoate (100 mg/mL; Cambridge Isotope Laboratories, Inc., Andover, MA, USA) was infused slowly over the course of 5 minutes into the stomach via the nasogastric tube. End-expiratory breath samples were obtained from the ventilation tube using a T-adapter (Datex-Engström, now part of GE Healthcare, Little Chalfont, Buckinghamshire, UK) and holder for vacutainers (blood needle holder; Reko Pty Ltd, Lisarow, Australia) containing a needle (VenoJect^®^; Terumo Corporation, Tokyo, Japan). Samples were collected at baseline, every 5 minutes for the first hour, and every 15 minutes thereafter, for a subsequent 3 hours after meal administration [33]. Time (t) = 0 minutes was defined as the time when all of the Ensure had been infused into the stomach. To avoid sampling other than end-expiratory air, sampling was timed to the end-expiratory phase by observation of the patient and the time-flow curve on the ventilation monitor.

Blood samples (5 mL) for the measurement of plasma CCK and PYY were collected into chilled EDTA (ethylenediaminetetraacetic acid) tubes immediately before and at 60 and 120 minutes after the delivery of the intragastric meal. Blood samples were centrifuged at 4°C within 30 minutes of collection and stored at -70°C for subsequent analysis. Blood samples for the measurement of blood glucose were also collected at baseline, every 15 minutes for the first hour, and every 30 minutes for the subsequent 3 hours.

### Measurements

#### Gastric emptying

GE was assessed indirectly by using ^13^C-octanoate breath tests. This non-invasive technique has been validated against gastric scintigraphy, using both solid and liquid meals, in healthy subjects and non-critically ill patients [34-39]. In critically ill patients, the breath test has a sensitivity of 71% and a specificity of 100% in detecting delayed GE, with a modest correlation between gastric half-emptying time determined by breath test and scintigraphy [40].

The concentration of CO_2 _and the percentage of ^13^CO_2 _were measured in each sample by means of an isotope ratio mass spectrometer (ABCA model 20/20; Europa Scientific, Crewe, UK). Samples containing less than 1% CO_2 _were regarded as being non-end-expiratory and were excluded from further analysis. The ^13^CO_2 _concentration over time was plotted, and the resultant curves were used to calculate a GE coefficient (GEC) [41], using non-linear regression formulae: GEC = ln(y)) and y = *at*^*b*^*e *^-*et*^, where y is the percentage of ^13^CO_2 _excretion in breaths per hour, t is time in hours, and a, b, and c are regression estimated constants [36,38,42]. GEC is a global index for the GE rate, and the normal range for normal GE has been established previously in a group of 28 healthy volunteers (normal GEC = 3.2 and 3.8) [33].

#### Plasma cholecystokinin, peptide YY, and blood glucose

Plasma CCK concentrations were measured by radioimmunoassay using an adaptation of the method of Santangelo and colleagues [43]. A commercially available antibody (C2581, lot 105H4852; Sigma-Aldrich, St. Louis, MO, USA) raised in rabbits against synthetic sulphated CCK-8 was used. This antibody binds to all CCK peptides containing the sulphated tyrosine residue in position 7 and has 26% cross-reactivity with un-sulphated CCK-8, less than 2% cross-reactivity with human gastrin 1, and no cross-reactivity with structurally unrelated peptides. Antibody was added at a dilution of 1:17,500, and iodine-125-labeled sulphated CCK-8 with Bolton-Hunter reagent (74 TBq/mmol; Amersham International, now part of GE Healthcare) was used as a tracer. Incubation proceeded for 7 days at 4°C. The antibody-bound fraction was separated by the addition of dextran-coated charcoal containing gelatin (0.015 g gelatin, 0.09 g dextran, and 0.15 g charcoal in 30 mL of assay buffer). The detection limit was 1 pmol/L, and the intra-assay coefficient of variation at 50 pmol/L was 9.5%.

Plasma PYY concentrations were measured by radioimmunoassay using an antiserum raised in rabbits against human PYY (1–36) (Sigma-Aldrich) [43]. This antiserum showed less than 0.001% cross-reactivity with human pancreatic polypeptide and sulphated CCK-8 and 0.0025% cross-reactivity with human neuropeptide Y. Tracer (Prosearch International, Malvern, Australia) was prepared by radio-labeling synthetic human PYY (1–36) (Auspep Pty Ltd, Parkville, Australia) using the lactoperoxidase method. Mono-iodo-tyrosine-PYY was separated from free iodine-125, diiodo-PYY, and unlabeled PYY by reverse-phase high-performance liquid chromatography (Phenomenex Jupiter C4 300A 5u column catalogue number 00B-4167-EO 250 _ 4.6 mm; Phenomenex, Inc., Torrance, CA, USA). Standards (1.6 to 50 fmol/tube) or samples (200 μL of plasma) were incubated in assay buffer with 100 μL of antiserum at a final dilution of 1:10,000 for 20 to 24 hours at 4°C, 100 μL of iodinated PYY (10,000 cpm) was then added, and the incubation continued for another 20 to 24 hours. Separation of the antibody-bound tracer from free tracer was achieved by the addition of 200 μL of dextran-coated charcoal containing gelatin (0.015 g of gelatin, 0.09 g of dextran, and 0.15 g of charcoal per 30 mL of assay buffer) and the mixture was incubated at 4°C for 20 minutes and then centrifuged at 4°C for 25 minutes. Radioactivity of the bound fraction was determined by counting the supernatants in a gamma counter. The intra- and inter-assay coefficients of variation were 12.3% and 16.6%, respectively. The minimum detectable concentration was 4 pmol/L [43]. Blood glucose concentrations were measured by means of a portable glucometer (Precision Plus; Abbott Laboratories, Abbott Park, IL, USA).

### Statistical analysis

Data are presented as mean ± standard error of the mean. The integrated changes in plasma concentrations of CCK and PYY were calculated and expressed as areas under the curve over the 120 minutes (AUC_0–120 min_) after the Ensure meal. Differences in demographic characteristics, in baseline blood glucose, CCK, and PYY concentrations, and in AUC_0–120 min _for plasma CCK and PYY between critically ill groups were compared using the Student unpaired *t *test and the chi-square test. Changes in plasma concentrations of CCK and PYY over time were determined by one-way repeated measures analysis of variance (ANOVA). Potential differences between patients with normal versus delayed GE with respect to the plasma CCK, PYY, and blood glucose responses to the meal were evaluated using two-way ANOVA with *post hoc *analyses. The relationships between GE with baseline plasma CCK and PYY, changes in plasma CCK and PYY (from baseline to t = 60 minutes and t = 120 minutes), and demographic factors (age, body mass index [BMI], Acute Physiology and Chronic Health Evaluation [APACHE] II score [41], and serum creatinine) were assessed using the Pearson correlation. Significance was accepted at a *P *value of less than 0.05.

## Results

The duration of ICU stay prior to the study was 4.60 ± 0.34 days. The admission diagnoses included multi-trauma (*n *= 12), head injury (*n *= 12), sepsis (*n *= 11), respiratory failure (*n *= 9), cardiac failure (*n *= 3), aortic dissection (*n *= 3), pancreatitis (*n *= 1), and retroperitoneal bleed (*n *= 1). The mean APACHE II score on the study day was 22.4 ± 0.9. Twenty-five patients (64%) were sedated with morphine and midazolam, and 14 patients (36%) with propofol. Nineteen patients (48%) required inotropic support with either adrenaline or noradrenalin. Acid suppression therapy (ranitidine or pantoprazole) was given to 32 (82%) of the 39 patients. Renal function was normal in the majority of patients (82%; 32/39) at the time of study, with a serum creatinine of 0.10 ± 0.01 mmol/L. None of the 7 patients with renal impairment (mean serum creatinine = 0.23 ± 0.04 mmol/L) required haemodialysis. Before enrolment into the study, 24 (66%) patients had received enteral feeds for a mean duration of 3.52 ± 0.36 days, and 15 (34%) patients had not received any nutritional support prior to the study. Ten patients (42%) who received prior enteral nutrition had feed intolerance, defined as aspirates of greater than 250 mL during gastric enteral feeding [44]. The mean duration of ICU stay prior to the study did not differ between the two groups (fed: 4.9 ± 0.5 days versus not fed: 4.2 ± 0.4 days; *P *= 0.78).

### Gastric emptying

GE was delayed in 64% (25/39) of the patients, with a mean GEC of 2.8 ± 0.1. The demographic data and characteristics of patients who had normal and delayed GE are summarised in Table 1. There was no relationship between the GEC and age (*P *= 0.23), gender (*P *= 0.82), BMI (*P *= 0.86), APACHE II score at time of study (*P *= 0.68), type of sedation, use of inotropes or acid suppression, presence of sepsis, or prior enteral feeding. The mean fasting blood glucose concentration was 7.14 ± 0.24 mmol/L, which increased slightly after the meal to a peak of 8.13 ± 0.28 mmol/L (*P *< 0.01). There were no differences in either fasting or postprandial blood glucose concentrations between patients with delayed and normal GE (*P *> 0.05).

**Table 1:** Demographic data and characteristics of critically ill patients, classified according to their rate of gastric emptying

	Normal GE (*n *= 14)	Delayed GE (*n *= 25)	*P *value
Age, years	57.5 ± 3.8	56.3 ± 2.8	0.87
Gender, male/female	7/7	17/8	0.41
Body mass index, kg/m^2^	28.3 ± 1.3	27.7 ± 1.2	0.78
APACHE II score on study day	22.6 ± 1.1	22.1 ± 1.0	0.86
Serum creatinine, mmol/L	0.08 ± 0.01	0.11 ± 0.02	0.14
Baseline blood glucose, mmol/L	7.1 ± 0.2	7.1 ± 0.2	0.99
Admission diagnosis^a^, percentage (number)			
Sepsis	36% (5)	19% (5)	0.28
Respiratory failure	43% (6)	15% (4)	0.13
Multi-trauma	21% (3)	32% (8)	0.48
Head injury^b^	21% (3)	34% (9)	0.48
Aortic dissection	7% (1)	8% (2)	0.99
Pancreatitis	0% (0)	4% (1)	0.99
Retroperitoneal bleed	7% (1)	0% (0)	0.35
Medication, percentage (number)			
Morphine ± midazolam	57% (8)	68% (17)	0.44
Propofol	43% (6)	31% (8)	0.44
Inotropes (adrenaline/noradrenalin)	57% (8)	46% (12)	0.51
Plasma CCK concentration, pmol/L			
Fasting	6.1 ± 0.4	8.5 ± 1.0	0.045
Postprandial			
At 60 minutes	8.2 ± 0.7	10.1 ± 0.8	0.03
At 120 minutes	7.1 ± 0.7	9.8 ± 0.8	0.03
Plasma PYY concentration, pmol/L			
Fasting	15.6 ± 1.3	22.8 ± 2.2	0.03
Postprandial			
At 60 minutes	21.0 ± 1.8	25.0 ± 2.2	0.02
At 120 minutes	18.9 ± 1.9	24.9 ± 2.0	0.02

Data are mean ± standard error of the mean. ^a^One patient may have one or more admission diagnoses. ^b^Including sub-arachnoid haemorrhage and massive cerebral ischemic event. APACHE II, Acute Physiology and Chronic Health Evaluation II; CCK, cholecystokinin; GE, gastric emptying; PYY, peptide YY.

### Plasma cholecystokinin and peptide YY concentrations

Baseline plasma CCK concentration was 7.74 ± 0.87 pmol/L and PYY was 20.4 ± 2.0 pmol/L. Baseline plasma PYY, but not CCK, was positively related to age (*r *= 0.37; *P *= 0.01) and BMI (*r *= 0.50; *P *< 0.01). Baseline plasma concentrations of both CCK and PYY were not related to gender (*P *= 0.82), the APACHE II score on the study day (*P *= 0.40), serum creatinine (*P *= 0.28), the type of sedation, the use of inotropes or acid suppression, the presence of sepsis, or prior enteral nutrition. There was no relationship between baseline plasma CCK and PYY (*P *= 0.80).

**Figure 1:**
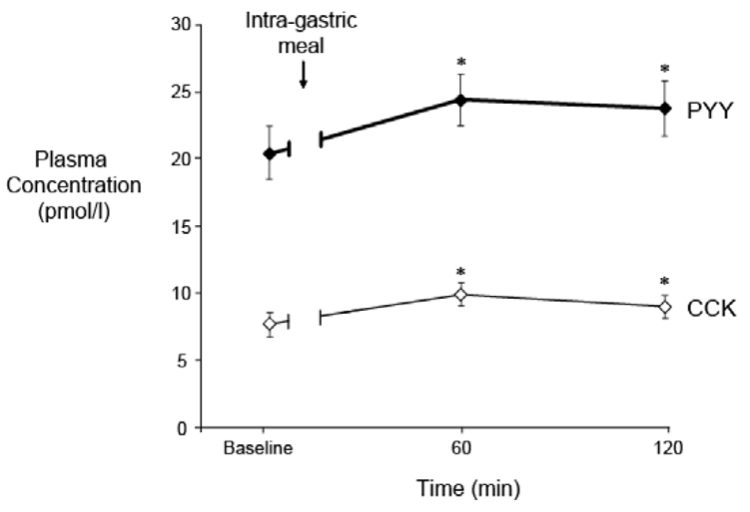
Plasma cholecystokinin (CCK) and peptide YY (PYY) concentrations at baseline and after intragastric Ensure (100 mL, 106 kcal with 21% lipid) in 39 critically ill patients (mean ± standard error of the mean). *P < 0.05 versus baseline.

In response to the gastric meal, there was a small but significant rise in plasma CCK and PYY (*P *= 0.01) (Figure 1). The integrated changes in plasma CCK (*r *= 0.45; *P *< 0.001), but not PYY, from baseline to 120 minutes were positively correlated with age. There was no relationship between integrated plasma CCK or PYY with gender, BMI, APACHE II scores on study day, serum creatinine, the type of sedation, the use of inotropes and acid suppression, presence of sepsis, or prior history of receiving enteral nutrition. Both plasma CCK and PYY remained above baseline at 120 minutes (Figure 1), particularly in patients with delayed GE (*P *< 0.05) (Table 1). There was a positive correlation between the magnitude of the increase in plasma PYY and CCK concentrations at 60 minutes (*r *= 0.33; *P *= 0.03).

### Relationship between gastric emptying, plasma cholecystokinin, and peptide YY

**Figure 2:**
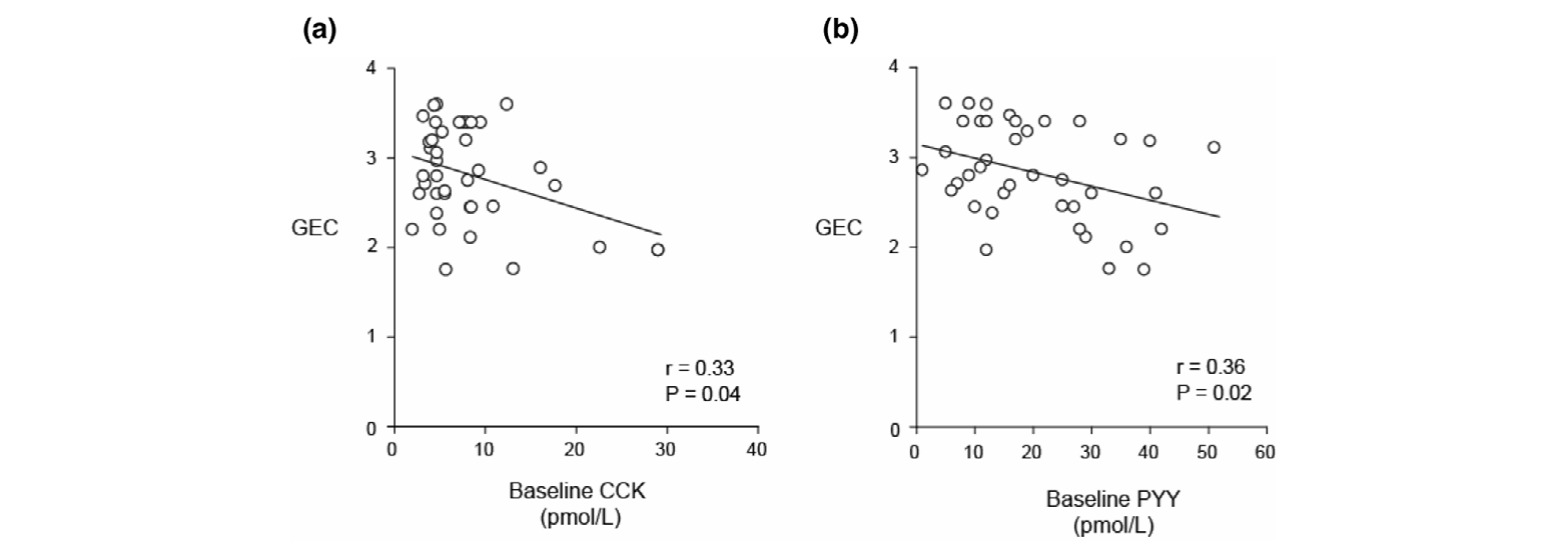
The relationship between the rate of gastric emptying, as assessed by the gastric emptying coefficient (GEC), and baseline plasma concentrations of cholecystokinin (CCK) **(a) **and peptide YY (PYY) **(b)**.

**Figure 3:**
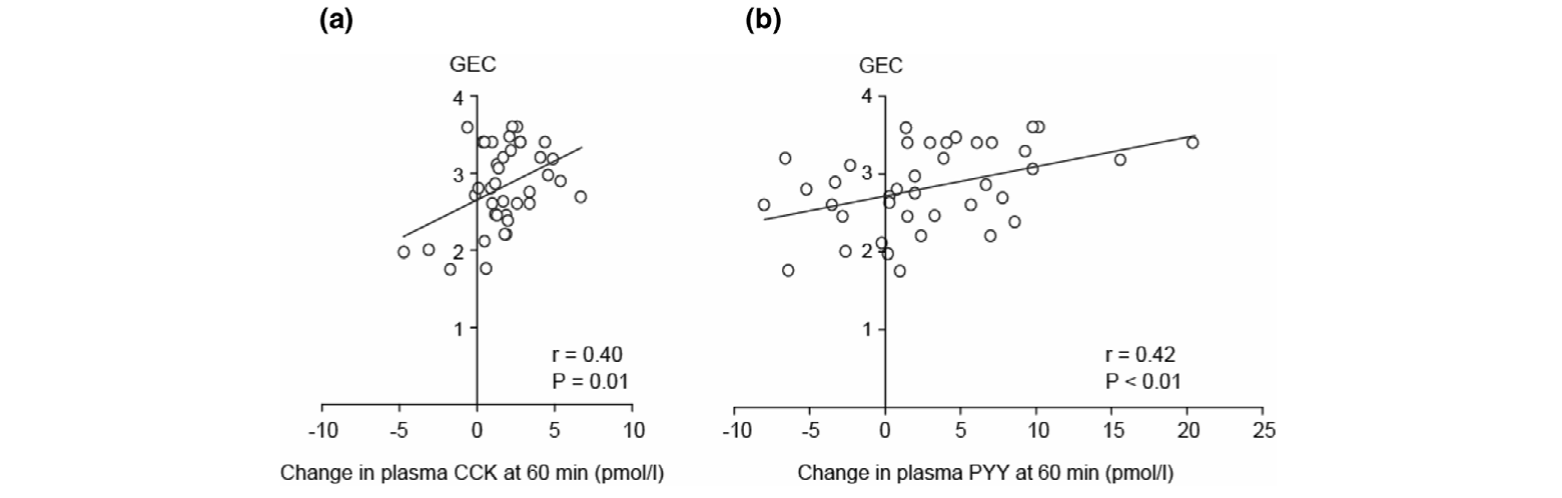
The relationship between the changes in plasma cholecystokinin (CCK) **(a) **and peptide YY (PYY) **(b) **from baseline to 60 minutes and the rate of gastric emptying, as assessed by the gastric emptying coefficient (GEC).

Baseline plasma CCK (8.5 ± 1.0 versus 6.1 ± 0.4 pmol/L; *P *= 0.045) and PYY (22.8 ± 2.2 versus 15.6 ± 1.3 pmol/L; *P *= 0.03) concentrations were higher in patients with delayed GE compared with those with normal GE. The GEC was inversely related to both baseline plasma CCK (*r *= -0.33; *P *= 0.04) and PYY (*r *= -0.36; *P *= 0.02) (Figure 2). Similarly, plasma CCK (*P *= 0.03) and PYY (*P *= 0.02) concentrations were higher at 60 and 120 minutes in patients with delayed GE. The GEC was inversely related to plasma CCK (*r *= -0.32; *P *= 0.049) and PYY (*r *= -0.30; *P *= 0.06) at 120 minutes, but not at 60 minutes. The absolute changes in plasma CCK (*r *= 0.40; *P *= 0.01) and PYY (*r *= 0.42; *P *< 0.01) at 60 minutes, as well as the integrated changes in plasma CCK (*r *= 0.36; *P *= 0.03) and PYY over 120 minutes (*r *= 0.38; *P *= 0.02), were directly related to the GEC (Figure 3). The integrated changes in plasma CCK and PYY, however, were not significantly different in patients with delayed versus normal GE (CCK: AUC _0–120 min_: 130 ± 42 versus 160 ± 38 pmol/L-minutes, *P *= 0.61; PYY: AUC _0–120 min_: 174 ± 98 versus 414 ± 155 pmol/L-minutes, *P *= 0.16).

## Discussion

Whilst we have shown previously that plasma CCK and PYY levels are increased in critically ill patients [28-30] and that CCK and PYY are known to slow GE, the present study is the first to directly demonstrate a relationship between GE and plasma concentrations of CCK and PYY in critical illness. The major observations are that, during critical illness, (a) GE was inversely related to both fasting and postprandial plasma CCK and PYY concentrations but (b) the postprandial increases in plasma CCK and PYY were also directly related to GE. Together with previous studies that have shown that enterogastric hormones [28-30] and feedback responses [27] to small intestinal nutrients are exaggerated in the critically ill, the relationship between enterogastric hormones and GE in the present study supports a putative pathogenesis role of enterogastric hormones in disordered GE during critical illness. However, the weakness of the relationship in these patients when compared with that previously reported in healthy subjects [1,2,4,7,11,12] highlights the complexity of the regulatory mechanisms and further suggests that other factors such as admission diagnosis and medication have a role in disordered GE.

The substantially higher fasting plasma CCK and PYY concentrations in our critically ill patients with delayed GE are consistent with our previous reports on critically ill patients with feed intolerance [28-30]. The observation that the rate of GE is inversely related to the fasting levels of CCK and PYY suggests that they may contribute to the regulation of GE in critically ill patients. Although the strength of the correlation was only modest, the relationship should not be regarded as weak, as this was a cross-sectional study. The mechanisms underlying the elevated fasting levels of these hormones are unknown. Nutritional deprivation is likely to be relevant since inadequate nutritional support is common in critically ill patients, fasting slows GE even in healthy subjects, and fasting CCK and PYY concentrations are higher in patients with anorexia nervosa and malnutrition [21,22]. The lack of differences in fasting hormonal concentrations between patients with and without nutritional support in the present study suggests that the duration of nutritional deprivation may have been insufficient for this effect to become apparent. Prolonged exposure of nutrients from previous feeds related to coexistent small intestinal hypo-motility [25,45] is an unlikely factor as all patients in the present cohort had been fasted for at least 8 hours. Renal impairment is also unlikely to contribute significantly to our observations as the proportion of patients with renal impairment was small and plasma CCK concentrations in this group did not differ from those with normal renal function. Although the majority of our patients received acid suppression therapy and therefore may have had increased serum gastrin levels, the cross-reactivity between gastrin and CCK is less than 2% [46] and is unlikely to contribute to the elevated CCK concentrations.

As observed in lean [1,2,4,7,11,12] and obese [47] healthy subjects, the present study demonstrates a weak but direct relationship between the rate of GE and postprandial increases in plasma CCK and PYY in critically ill patients. This indicates that the release of these hormones is dependent on the amount of nutrient delivered into the small intestine, particularly fat [48]. This observation is at variance with recent findings that suggest that critically ill patients with feed intolerance have higher plasma CCK and PYY release in response to duodenal nutrients than patients who tolerated feeds [28,29]. The reason for the apparent discrepancy is unknown but may relate to the difference in the site of nutrient administration. Given the relatively small gastric nutrient load and high frequency of delayed GE (64%), the existence of the relationship between GE and the hormonal release may simply reflect the level of duodenal nutrient stimulation. This possibility is supported by the observation that the hormonal release in the present study was small and similar to that seen in patients who received duodenal nutrition stimulation during 1 kcal/minute [28,29]. However, the concept of heightened hormonal release from a similar nutrient load in critically ill patients, particularly those with impaired motility [28,29], is also illustrated in the present study by the finding that the overall increase in plasma CCK and PYY in patients with delayed GE was similar to those with normal GE. Therefore, in patients with delayed GE, although only a small amount of nutrient was delivered into the duodenum, the 'increased sensitivity' of the duodenal receptors leads to a greater hormonal release for the given nutrient load. Together, these findings suggest that, in critically ill patients, there is a complex interaction between GE, intestinal nutrients, and hormonal release.

Consistent with our recent study [29], the postprandial release of PYY is related to the release of CCK, which supports the concept that CCK is an important proximal mediator for the release of PYY [9,10]. Furthermore, evidence from animal studies [49] suggests that PYY may be released by direct neural stimulation from nutrients in the proximal intestine, possibly via a neural linkage between the proximal gut to the distal PYY-secreting cells which involves sensory vagal fibres with nicotinic, beta-adrenergic, opioid, and serotonergic synapses and nitric oxide release [49,50].

Although blood glucose concentrations were adequately controlled by the standardized insulin therapy, the potential impact of insulin on the enterogastric feedback and the hormonal release is not known. Whilst insulin-induced hypoglycaemia has no significant effect on antro-pyloro-duodenal motor activity in humans [51], it accelerates GE [52]. Currently, there are no data on the impact of insulin on the release of CCK or PYY in humans. In the present study, the effects of insulin on the enterogastric feedback and the hormonal release are likely to be small as all patients received the therapy. In addition, insulin is essential in this study as it minimized the adverse impact of hyperglycaemia on the rate of GE.

Whilst the present observations strengthen the rationale for the potential use of CCK antagonists in the management of feed intolerance in the critically ill, it should be recognised that the efficacy of such agents may be limited due to the complex interaction amongst many factors that regulate GE during critical illness. Despite this reservation, loxiglumide (a CCK antagonist) has been shown to accelerate the GE of lipid-rich liquid meals in healthy subjects [17-19,53] as well as patients with functional dyspepsia [54] and irritable bowel syndrome [55]. Currently, PYY antagonists are not available for use in humans.

## Conclusion

During critical illness, the relationship between GE, plasma CCK, and PYY is complex. Whilst GE is inversely related to fasting and postprandial plasma CCK and PYY concentrations, it may also be a determinant of CCK and PYY response to a meal. The role of these enterogastric hormones in the pathogenesis of impaired GE during critical illness, however, requires further evaluation with specific antagonists.

## Key messages

• Although enterogastric feedback in response to nutrient is enhanced and plasma concentrations of both cholecystokinin (CCK) and peptide YY (PYY) are elevated in critically ill patients, the relationships between plasma CCK and PYY concentrations and gastric emptying (GE) have not been evaluated in these patients.

• This study is the first to establish the complex relationship between GE and plasma concentrations of CCK and PYY in critical illness.

• The major observations of this study are that during critical illness:

i. plasma CCK and PYY concentrations were higher in patients with delayed GE, during both fasting and for 2 hours postprandially, and

ii. GE was inversely related to both fasting and postprandial plasma CCK and PYY concentrations, but the postprandial increases in plasma CCK and PYY were also directly related to GE.

• Whilst these findings support the potential role of these enterogastric hormones in the pathogenesis of impaired GE during critical illness, further evaluation with specific antagonists is warranted.

## Abbreviations

ANOVA = analysis of variance; APACHE = Acute Physiology and Chronic Health Evaluation; AUC_0–120 min _= area under curve over the course of 120 minutes; BMI = body mass index; CCK = cholecystokinin; GE = gastric emptying; GEC = gastric emptying coefficient; ICU = intensive care unit; PYY = peptide YY.

## Competing interests

The authors declare that they have no competing interests.

## Authors' contributions

NQN participated in the study design, carried out the studies and performed data and statistical analysis, helped draft the manuscript, and was involved in recruiting patients from the ICU of the Royal Adelaide Hospital. RJF, RHH, and MH helped conceive the study, participated in its design and coordination, and helped draft the manuscript. LKB helped carry out the studies and collection of data and helped draft the manuscript. MJC helped conceive the study, participated in its design and coordination, and was involved in recruiting patients from the ICU of the Royal Adelaide Hospital. JW performed the radioimmunoassay. RB was responsible for analysing GE breath samples. All authors read and approved the final manuscript.
